# Supplementary material for: Gut microbiome remodeling by antibiotics reduces neuroinflammation in traumatic brain injury
Source: Res Sq. 2025 Sep 18:rs.3.rs-7411086. Preprint. [Version 1] doi: 10.21203/rs.3.rs-7411086/v1 (PMC12458575; doi:10.21203/rs.3.rs-7411086/v1)
Supplement: Supplement 1 [file NIHPPrs7411086v1-supplement-1.pdf]

**Supplemental Table 1.**

| REAGENT or RESOURCE | SOURCE | IDENTIFIER |
|---|---|---|
| **Antibodies** | | |
| anti-rabbit Iba-1 | Wako | CAT#019-19741; RRID: AB_839504 |
| anti-mouse CD68 | Bio-Rad | CAT#019-19741; RRID: AB_839504 |
| anti-rat F4/80 | R&D Systems | CAT#MAB5580 |
| anti-rabbit P2Y12 | Anaspec | CAT#AS-55042A; RRID: AB_2267540 |
| anti-rabbit GFAP | Millipore | CAT#MAB360 |
| anti-rat Ly6B2 | Bio-Rad | CAT#MCA771GA; RRID: AB_324243 |
| Anti-rabbit Alexa Fluor 568-conjugated IgG secondary antibody | Thermo Fisher Scientific | CAT#11036 |
| anti-rat Alexa Fluor 488-conjugated IgG secondary antibody | Thermo Fisher Scientific | CAT#A11006 |
| anti-rabbit Alexa Fluor 488-conjugated IgG secondary antibody | Thermo Fisher Scientific | CAT#A11008 |
| Anti-mouse Alexa Fluor 568-conjugated IgG secondary antibody | Thermo Fisher Scientific | CAT#A11031 |
| DAPI solution | Sigma-Aldrich | CAT#62248 |
| **Chemicals, peptides, and recombinant proteins** | | |
| Ampicillin sodium | Goldbio | CAT#A-301-10 |
| Vancomycin hydrochloride | Goldbio | CAT#V-200-5 |
| Gentamicin sulfate | Goldbio | CAT#G-400-5 |
| Metronidazole | Spectrum | CAT#M1511-25GM |
| 0.1% cresyl-violet solution | Sigma-Aldrich | CAT#AAJ6431809 |
| Permount | ThermoFisher | CAT#SP15100 |
| Normal Goat Serum (NGS) | Vector Laboratories | CAT#NC9270494 |
| Fluoro-gel with Tris Buffer | Fisher Science | CAT#5024704 |
| Hematoxylin with Acetic Acid | Fisher Science | CAT#NC9064721 |
| Eosin Phloxine Stain | Fisher Science | CAT#NC9126775 |
| G-Biosciences Nuclear Fast Red Solution | Fisher Science | CAT#AAJ6012209 |
| Alcian Blue 8GX | Fisher Science | CAT#50-134-4520 |
| **Critical commercial assays** | | |
| Red Assay RNAscope | Advanced Cell Diagnostics Inc. | CAT#322360 |
| In Situ Cell Death Detection Kit, Fluorescein | Sigma-Aldrich | CAT#11684795910 |
| QIAamp PowerFecal Pro DNA Kit | Qiagen | CAT#51804 |
| **Deposited data** | | |
| 16S RNA sequencing | SRA BioProject | PRJNA1104663 |
| Metagenomic sequencing | SRA BioProject | PRJNA1216228 |
| High-quality metagenome-assembled genomes | BioSample | SAMN45875152 - SAMN45875164 |
| **Experimental models: Organisms/strains** | | |
| Mice: C57BL/6J | Jackson Laboratory | Strain no. JAX: 000664 |
| Mice: C57BL/6-GF (Germ Free) | Baylor College of Medicine Gnotobiotic Rodent Facility | |

| | | |
|---|---|---|
| **Oligonucleotides** | | |
| RNAscope Probe Mm-TNFa | Advanced Cell Diagnostics Inc. | CAT#311081 |
| RNAscope Probe Mm-IL6 | Advanced Cell Diagnostics Inc. | CAT#315891 |
| **Software and algorithms** | | |
| Neurolucida Morphometric | MBF Biosciences | https://www.mbfbioscience.com/products/neurolucida/ |
| Image J | NIH | https://imagej.net/ij/ |
| NeuroExplorer | MBP Biosciences | https://www.neuroexplorer.com/ |
| GraphPad Prism 8 | GraphPad | https://www.graphpad.com/ |
| MetaboAnalystR | MetaboAnalyst | https://www.metaboanalyst.ca/ |
| Custom R and bash code | Villapol Lab Github | https://github.com/villapollab/adult_abx_tbi |
| **Other** | | |
| Leica Impact One Stereotaxic Impactor | Leica Biosystems | RRID:SCR_025114 |
| FastPrep-24 system | MP Biomedicals | CAT#116004500 |
| DS-11 Series Spectrophotometer/Fluorometer | DeNovix | |
| Rotarod | Ugo Basile | CAT#47650 |
| Nikon fluorescence microscope Eclipse Ni-U | Nikon Instruments Inc. | |
| Confocal Imaging System | Leica Microsystems | |
| Epredia Cryostat NX50 | Fisher Scientific | |

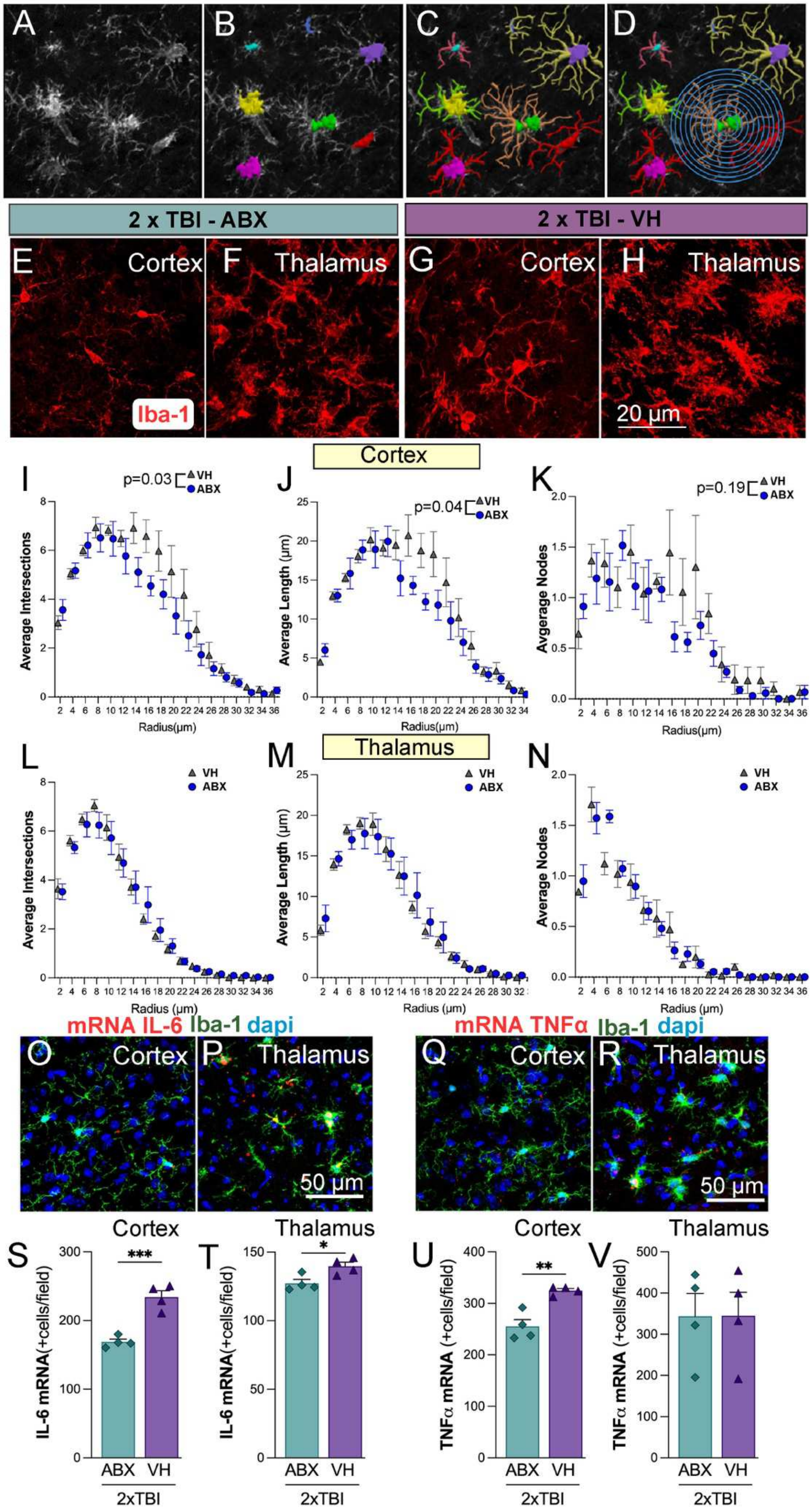

A
B
C
D
2 x TBI - ABX
2 x TBI - VH
E
Cortex
F
Thalamus
G
Cortex
H
Thalamus
Iba-1
20 µm
Cortex
I
p=0.03
VH
ABX
Average Intersections
Radius(µm)
J
p=0.04
VH
ABX
Average Length (µm)
Radius(µm)
K
p=0.19
VH
ABX
Avgerage Nodes
Radius(µm)
Thalamus
L
VH
ABX
Average Intersections
Radius (µm)
M
VH
ABX
Average Length (µm)
Radius (µm)
N
VH
ABX
Average Nodes
Radius (µm)
mRNA IL-6 Iba-1 dapi
O
Cortex
P
Thalamus
50 µm
mRNA TNFα Iba-1 dapi
Q
Cortex
R
Thalamus
50 µm
Cortex
Thalamus
Cortex
Thalamus
S
***
IL-6 mRNA(+cells/field)
ABX
VH
2xTBI
T
*
IL-6 mRNA(+cells/field)
ABX
VH
2xTBI
U
**
TNFα mRNA (+cells/field)
ABX
VH
2xTBI
V
TNFα mRNA (+cells/field)
ABX
VH
2xTBI

**Supplementary Figure 1. Microglial morphological changes following repeated TBI with and without ABX treatment.** (A-D) Representative images of microglial morphology analysis in the 2xTBI-VH group. (A) Original grayscale Iba-1 staining image, (B) segmented microglia with color-coded individual cells, (C) skeletonized microglia used for Sholl analysis, and (D) overlay of Sholl analysis with concentric circles to assess microglial branching complexity. (E-H) Representative Iba-1 immunofluorescence images of microglia in the cortex (E, G) and thalamus (F, H) from 2xTBI-ABX and 2xTBI-VH groups, respectively. Increased microglial activation is observed in the VH-treated group compared to the ABX-treated group. (I-N) Sholl analysis quantifying microglial morphology in the cortex (I-K) and thalamus (L-N). (I, L) Quantifying average intersections of microglial processes across radial distances from the soma shows a significant reduction in the ABX group compared to VH in the cortex. (J, M) The average process length decreases in the ABX-treated group in the cortex ($p = 0.04$) but not in the thalamus. (K, N) Average number of branch nodes, with no significant differences observed between groups. (O-R) Representative images of in situ hybridization for pro-inflammatory cytokines IL-6 (O, P) and TNFα (Q, R) in microglia (Iba-1, green) with nuclear counterstain (DAPI, blue), showing increased expression in the VH-treated group compared to ABX. (S, T) IL-6 mRNA+ cell counts were significantly higher in the 2xTBI-VH group than in ABX in both the cortex and thalamus. (U, V) TNFα mRNA+ cell counts were also elevated in the cortex of the VH group ($p < 0.01$), though no significant differences were found in the thalamus. These results suggest that ABX treatment mitigates microglial activation and neuroinflammatory responses following repeated TBI, as evidenced by reduced microglial complexity and lower pro-inflammatory cytokine expression.

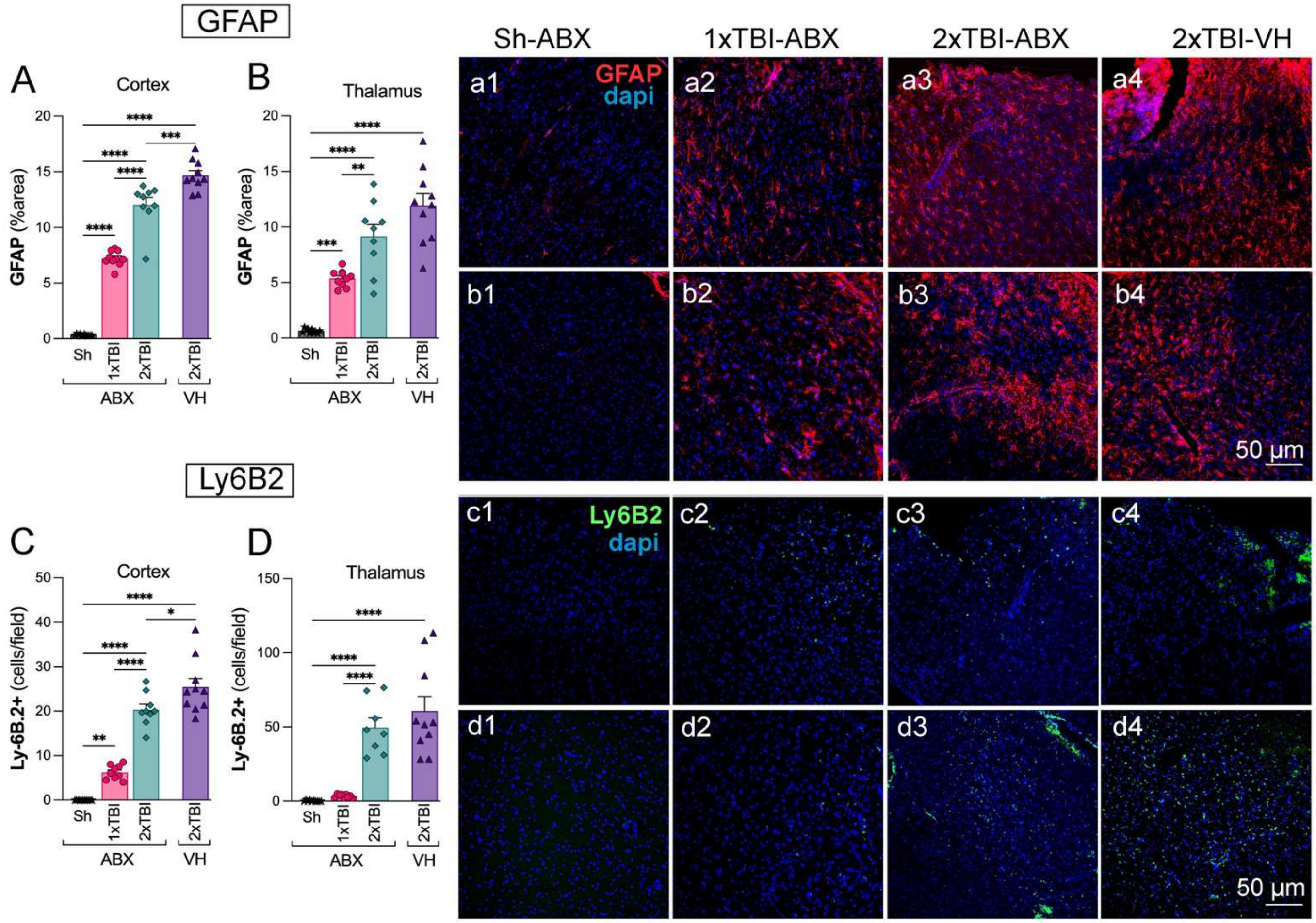


**Supplementary Figure 2. Antibiotic treatment decreases astrocyte reactivity but does not affect neutrophil infiltration following TBI.** (A, B) Quantification of GFAP+ staining (% area) in the cortex (A) and thalamus (B) of Sh-ABX, 1xTBI-ABX, 2xTBI-ABX, and 2xTBI-VH groups. (a1–a4, b1–b4) Representative GFAP immunofluorescence images (red) with DAPI nuclear staining (blue) in the cortex (a1–a4) and thalamus (b1–b4) of the respective groups. (C, D) Quantification of Ly6B2+ immune cells (cells/field) in the cortex (C) and thalamus (D) of Sh-ABX, 1xTBI-ABX, 2xTBI-ABX, and 2× TBI-VH groups (c1–c4, d1–d4). Representative immunofluorescence images for Ly6B2 (green) with DAPI nuclear staining (blue) in the cortex (c1–c4) and thalamus (d1–d4) across groups. Statistical significance: *p < 0.05, **p < 0.01, ***p < 0.001, ****p < 0.0001 (one-way ANOVA with Tukey's post hoc test) (n=10-9/group). Scale bar: 50 μm.

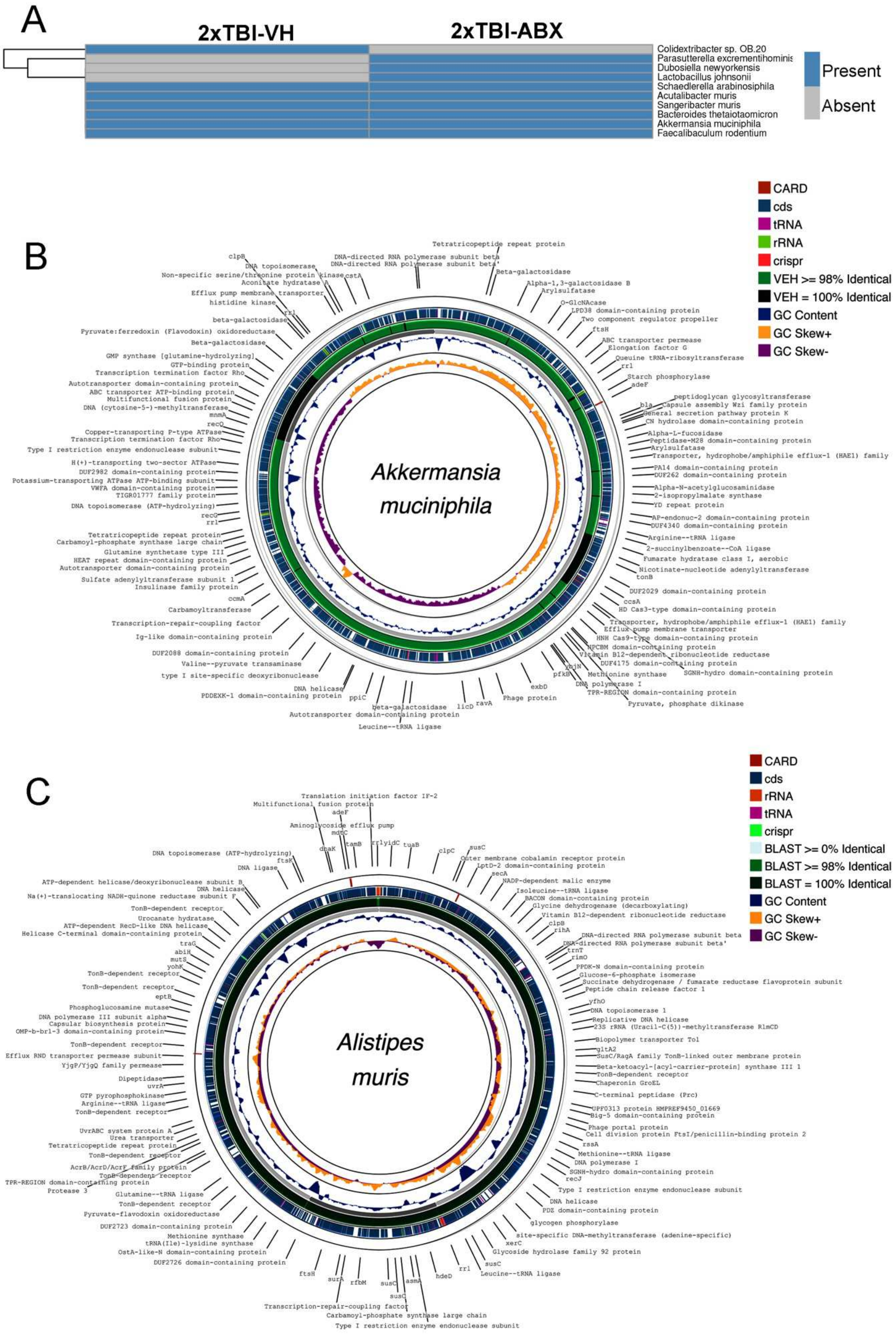
A
2xTBI-VH
2xTBI-ABX
Colidextribacter sp. OB.20
Parasutterella excrementihominis
Dubosiella newyorkensis
Lactobacillus johnsonii
Schaedlerella arabinosiphila
Acutalibacter muris
Sangeribacter muris
Bacteroides thetaiotaomicron
Akkermansia muciniphila
Faecalibaculum rodentium
Present
Absent
B
CARD
cds
tRNA
rRNA
crispr
VEH >= 98% Identical
VEH = 100% Identical
GC Content
GC Skew+
GC Skew-
Akkermansia muciniphila
Tetratricopeptide repeat protein
DNA-directed RNA polymerase subunit beta
DNA-directed RNA polymerase subunit beta'
Beta-galactosidase
Alpha-1,3-galactosidase B
Arylsulfatase
O-GlcNAcase
LPD38 domain-containing protein
Two component regulator propeller
ftsH
ABC transporter permease
Elongation factor G
Queuine tRNA-ribosyltransferase
rrl
Starch phosphorylase
adeF
peptidoglycan glycosyltransferase
bla
Capsule assembly Wzi family protein
General secretion pathway protein K
CN hydrolase domain-containing protein
Alpha-L-fucosidase
Peptidase-M28 domain-containing protein
Arylsulfatase
Transporter, hydrophobe/amphiphile efflux-1 (HAE1) family
PA14 domain-containing protein
DUF262 domain-containing protein
Alpha-N-acetylglucosaminidase
2-isopropylmalate synthase
YD repeat protein
AP-endonuc-2 domain-containing protein
DUF4340 domain-containing protein
Arginine--tRNA ligase
2-succinylbenzoate--CoA ligase
Fumarate hydratase class I, aerobic
Nicotinate-nucleotide adenylyltransferase
tonB
DUF2029 domain-containing protein
ccsA
HD Cas3-type domain-containing protein
Transporter, hydrophobe/amphiphile efflux-1 (HAE1) family
Efflux pump membrane transporter
HNH Cas9-type domain-containing protein
HPCBM domain-containing protein
Vitamin B12-dependent ribonucleotide reductase
DUF4175 domain-containing protein
SGNH-hydro domain-containing protein
ybjN
Methionine synthase
pfkB
DNA polymerase I
TPR-REGION domain-containing protein
Pyruvate, phosphate dikinase
exbD
Phage protein
ravA
licD
beta-galactosidase
Autotransporter domain-containing protein
Leucine--tRNA ligase
ppiC
DNA helicase
PDDEXK-1 domain-containing protein
type I site-specific deoxyribonuclease
Valine--pyruvate transaminase
DUF2088 domain-containing protein
Ig-like domain-containing protein
Transcription-repair-coupling factor
Carbamoyltransferase
ccmA
Insulinase family protein
Sulfate adenylyltransferase subunit 1
Autotransporter domain-containing protein
HEAT repeat domain-containing protein
Glutamine synthetase type III
Carbamoyl-phosphate synthase large chain
Tetratricopeptide repeat protein
rrl
recG
DNA topoisomerase (ATP-hydrolyzing)
TIGR01777 family protein
VWFA domain-containing protein
Potassium-transporting ATPase ATP-binding subunit
DUF2982 domain-containing protein
H(+)-transporting two-sector ATPase
Type I restriction enzyme endonuclease subunit
Transcription termination factor Rho
Copper-transporting P-type ATPase
recQ
mnmA
DNA (cytosine-5-)-methyltransferase
Multifunctional fusion protein
ABC transporter ATP-binding protein
Autotransporter domain-containing protein
Transcription termination factor Rho
GTP-binding protein
GMP synthase [glutamine-hydrolyzing]
Beta-galactosidase
Pyruvate:ferredoxin (Flavodoxin) oxidoreductase
beta-galactosidase
rrl
histidine kinase
Efflux pump membrane transporter
Aconitate hydratase A
Non-specific serine/threonine protein kinase
cstA
DNA topoisomerase
clpB
C
CARD
cds
rRNA
tRNA
crispr
BLAST >= 0% Identical
BLAST >= 98% Identical
BLAST = 100% Identical
GC Content
GC Skew+
GC Skew-
Alistipes muris
Translation initiation factor IF-2
Multifunctional fusion protein
adeF
Aminoglycoside efflux pump
mdtC
dnaK
tamB
rrl
yidC
tuaB
clpC
susC
Outer membrane cobalamin receptor protein
LptD-2 domain-containing protein
secA
NADP-dependent malic enzyme
Isoleucine--tRNA ligase
BACON domain-containing protein
Glycine dehydrogenase (decarboxylating)
Vitamin B12-dependent ribonucleotide reductase
clpB
rihA
DNA-directed RNA polymerase subunit beta
DNA-directed RNA polymerase subunit beta'
trnT
rimO
PPDK-N domain-containing protein
Glucose-6-phosphate isomerase
Succinate dehydrogenase / fumarate reductase flavoprotein subunit
Peptide chain release factor 1
yfhO
DNA topoisomerase 1
Replicative DNA helicase
23S rRNA (Uracil-C(5))-methyltransferase RlmCD
Biopolymer transporter Tol
gltA2
SusC/RagA family TonB-linked outer membrane protein
Beta-ketoacyl-[acyl-carrier-protein] synthase III 1
TonB-dependent receptor
Chaperonin GroEL
C-terminal peptidase (Prc)
UPF0313 protein HMPREF9450_01669
Big-5 domain-containing protein
Phage portal protein
Cell division protein FtsI/penicillin-binding protein 2
rssA
Methionine--tRNA ligase
DNA polymerase I
SGNH-hydro domain-containing protein
recJ
Type I restriction enzyme endonuclease subunit
DNA helicase
PDZ domain-containing protein
glycogen phosphorylase
site-specific DNA-methyltransferase (adenine-specific)
xerC
Glycoside hydrolase family 92 protein
susC
Leucine--tRNA ligase
rrl
hdeD
asmA
susC
susC
rfbM
surA
ftsH
Transcription-repair-coupling factor
Carbamoyl-phosphate synthase large chain
Type I restriction enzyme endonuclease subunit
DUF2726 domain-containing protein
OstA-like-N domain-containing protein
tRNA(Ile)-lysidine synthase
Methionine synthase
DUF2723 domain-containing protein
Pyruvate-flavodoxin oxidoreductase
TonB-dependent receptor
Glutamine--tRNA ligase
Protease 3
TPR-REGION domain-containing protein
TonB-dependent receptor
AcrB/AcrD/AcrF family protein
TonB-dependent receptor
Tetratricopeptide repeat protein
Urea transporter
UvrABC system protein A
TonB-dependent receptor
Arginine--tRNA ligase
GTP pyrophosphokinase
uvrA
Dipeptidase
YjgP/YjgQ family permease
Efflux RND transporter permease subunit
TonB-dependent receptor
OMP-b-brl-3 domain-containing protein
Capsular biosynthesis protein
DNA polymerase III subunit alpha
Phosphoglucosamine mutase
eptB
TonB-dependent receptor
TonB-dependent receptor
yohK
mutS
abiH
traG
Helicase C-terminal domain-containing protein
ATP-dependent RecD-like DNA helicase
Urocanate hydratase
TonB-dependent receptor
Na(+)-translocating NADH-quinone reductase subunit F
DNA helicase
ATP-dependent helicase/deoxyribonuclease subunit B
DNA ligase
ftsK
DNA topoisomerase (ATP-hydrolyzing)

**Supplementary Figure 3.** Metagenomic analysis reveals differential microbial composition and functional profiles in response to TBI and ABX treatment. (A) Presence-absence heatmap showing bacterial taxa identified in fecal samples from 2xTBI-VH and 2xTBI-ABX groups. Several bacterial species, including *Akkermansia muciniphila*, *Parasutterella excrementihominis*, and *Dubosiella newyorkensis*, were exclusively present or enriched in the VH-treated group. At the same time, other taxa, such as *Schaedlerella arabinosiphila* and *Sanguibacter muris*, were detected only in the ABX-treated group. (B) Circular genome visualization of *Akkermansia muciniphila*, highlighting annotated functional elements such as resistance genes (CARD), coding sequences (cds), transfer RNAs (tRNA), ribosomal RNAs (rRNA), and clustered regularly interspaced short palindromic repeats (CRISPR). The green rings indicate the genomic content comparison between the VH and ABX groups based on BLAST identity thresholds (≥0%, ≥98%, 100%). GC content and GC skew patterns are displayed in the innermost tracks, providing insights into potential functional adaptations under TBI conditions. (C) Circular genome representation of *Alistipes muris*, displaying key functional genes and genomic features as described for *Akkermansia muciniphila*, including transporters, antibiotic resistance genes, and metabolic regulators. Comparative genomic analysis reveals differences in gene presence and identity between the VH and ABX groups, suggesting functional adaptations in response to TBI and ABX treatment. These findings indicate distinct gut microbial compositions and genomic adaptations in response to TBI and antibiotic interventions, highlighting potential functional contributions of specific taxa to host-microbiome interactions post-injury.
